# Using Image Analysis Technique for Predicting Light Lamb Carcass Composition

**DOI:** 10.3390/ani14111593

**Published:** 2024-05-28

**Authors:** João J. Afonso, Mariana Almeida, Ana Catharina Batista, Cristina Guedes, Alfredo Teixeira, Severiano Silva, Virgínia Santos

**Affiliations:** 1 Centre for Interdisciplinary Research in Animal Health (CIISA), Faculty of Veterinary Medicine, University of Lisbon, Avenida da Universidade Técnica, 1300-477 Lisboa, Portugal; jafonso@fmv.ulisboa.pt; 2 Associate Laboratory of Animal and Veterinary Science (AL4AnimalS), Veterinary and Animal Research Centre (CECAV), University of Trás-os-Montes e Alto Douro, Quinta de Prados, 5000-801 Vila Real, Portugal; catharina.batista@gmail.com (A.C.B.); cguedes@utad.pt (C.G.); ssilva@utad.pt (S.S.); vsantos@utad.pt (V.S.); 3 Mountain Research Centre (CIMO), Escola Superior Agrária, Instituto Politécnico de Bragança, Campus Sta Apolónia Apt 1172, 5301-855 Bragança, Portugal; teixeira@ipb.pt

**Keywords:** video image analysis, light lambs, carcass composition

## Abstract

**Simple Summary:**

To categorize carcasses into value classes aligning with consumer preferences and to guarantee equitable compensation for producers, as well as to furnish valuable data for scientific research, numerous technologies have been devised to assess carcass composition with ever-increasing accuracy and precision. One such technology is video image analysis (VIA), which has demonstrated promising outcomes, particularly in its application to cattle and sheep carcasses. This study focuses on employing a VIA methodology for evaluating light lamb carcasses, a segment that has received comparatively less attention from both research and industry.

**Abstract:**

Over the years, numerous techniques have been explored to assess the composition and quality of sheep carcasses. This study focuses on the utilization of video image analysis (VIA) to evaluate the composition of light lamb carcasses (4.52 ± 1.34 kg, mean cold carcass weight ± SD). Photographic images capturing the lateral and dorsal sides of fifty-five light lamb carcasses were subjected to analysis. A comprehensive set of measurements was recorded, encompassing dimensions such as lengths, widths, angles, areas, and perimeters, totaling 21 measurements for the lateral view images and 29 for the dorsal view images. K-Folds stepwise multiple regression analyses were employed to construct prediction models for carcass tissue weights (including muscle, subcutaneous fat, intermuscular fat, and bone) and their respective percentages. The most effective prediction equations were established using data from cold carcass weight (CCW) and measurements from both dorsal and lateral views. These models accounted for a substantial portion of the observed variation in the weights of all carcass tissues (with K-fold-R^2^ ranging from 0.83 to 0.98). In terms of carcass tissue percentages, although the degree of variation explained was slightly lower (with K-fold-R^2^ ranging from 0.41 to 0.78), the VIA measurements remained integral to the predictive models. These findings underscore the efficacy of VIA as an objective tool for assessing the composition of light lamb carcasses, which are carcasses weighing ≈ 4–8 kg.

## 1. Introduction

Scholz et al. [1] pointed out that accurate and precise evaluation of body or carcass composition is important for performance testing, grading, and the selection or payment of meat-producing animals, as well as for scientific studies on growth, nutrition, genetics, housing, and behavior or farm animal well-being. Sorting carcasses into value classes based on consumer preferences, producers can be rewarded for delivering carcasses that meet industry and consumer requirements [2,3,4]) and, feeding this information back from the abattoir to the producers and breeders, it can be used in genetic evaluations, what will increase the accuracy of estimated breeding values and rates of response to selection [5]. However, direct evaluation of carcass composition implies dissection and/or chemical analyses, which are destructive, time-consuming, expensive, and prone to error due to human fatigue [6], while traditional indirect techniques based on linear measures have shown poor prediction accuracy [7,8]. So, in the process of developing new technologies to assess carcass composition with increasing accuracy and precision, preference has been given to non-invasive procedures, relying mainly on the development of electronic and computer-based techniques in order to provide objective phenotypic data [1]. Since the Kansas State University won, in 1980, a project to develop a prototype VIA [9], several works have been carried out to understand and develop the potential of this technology as a non-destructive nor invasive alternative to allow an objective, quick and precise evaluation of the carcass without interfering with the production chain [10]. The evaluation of carcasses with the VIA systems is based on measurements of length, width, area, angle, volume, and color made on images obtained during processing operations. Such measurements can then be used to estimate traits such as carcass yield, lean meat yield, saleable meat yield, yield in pieces, and percentage of fat or carcass muscularity [11,12,13,14]. The objective of the present study was to evaluate the potential of a VIA technology to estimate the tissue composition of light lamb carcasses, developing prediction equations for carcass muscle, fat, and bone.

## 2. Materials and Methods

### 2.1. Animals and Carcasses

Light carcasses of the fifty-five lambs from the indigenous Portuguese Churra da Terra Quente (CTQ) breed, in accordance with the Borrego Terrincho–PDO specifications [15], were selected for this. Briefly, the Borrego Terrincho–PDO lamb comes from the Churra da Terra Quente breed in Northeast Portugal and is primarily bred for milk production and milk-fed lambs. These lambs are typically slaughtered at 4–6 weeks of age and yield carcasses weighing approximately 4–8 kg. European meat quality labels, such as “Borrego Terrincho”–PDO, are linked to specific regions and traditional production methods, providing a level of uniqueness. The animals were processed in an accredited slaughterhouse, adhering strictly to both National and European regulations. Following slaughter, the carcasses underwent refrigeration at 4 °C for 24 h, during which the cold carcass weight (CCW) was recorded (4.52 ± 1.34 kg, mean CCW ± SD).

### 2.2. Acquisition of VIA Images and Measurements

Photographic images capturing both the dorsal and left outer side of each carcass were obtained. For the left outer side images, carcasses were suspended against a black background and repositioned for dorsal images; precautions were taken to stabilize the carcasses prior to image capture. A Nikon D3100 digital camera (Nikon Inc., Tokyo, Japan) equipped with an 8-megapixel sensor was used for image acquisition. The camera settings were manually configured: shutter speed at 1/60 s, aperture at F/4.5, ISO sensitivity set to 400, no flash, and a focal length of 26 mm. Images were saved in JPEG format. The entire process was conducted under consistent artificial lighting conditions and a fixed camera position, with the camera positioned 3 m from the carcasses.

**Figure 1:**
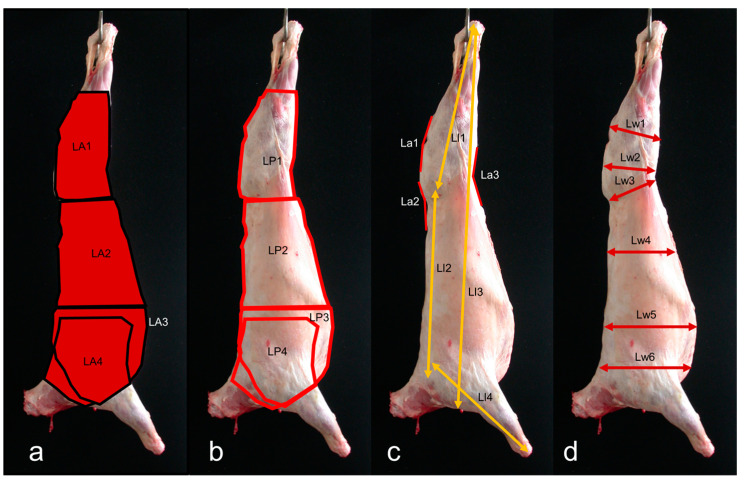
Lateral side view of light lamb carcass showing areas (**a**), perimeters (**b**), lengths and angles (**c**), and widths (**d**) measurements. For a brief measurement description please see Table 1.

**Figure 2:**
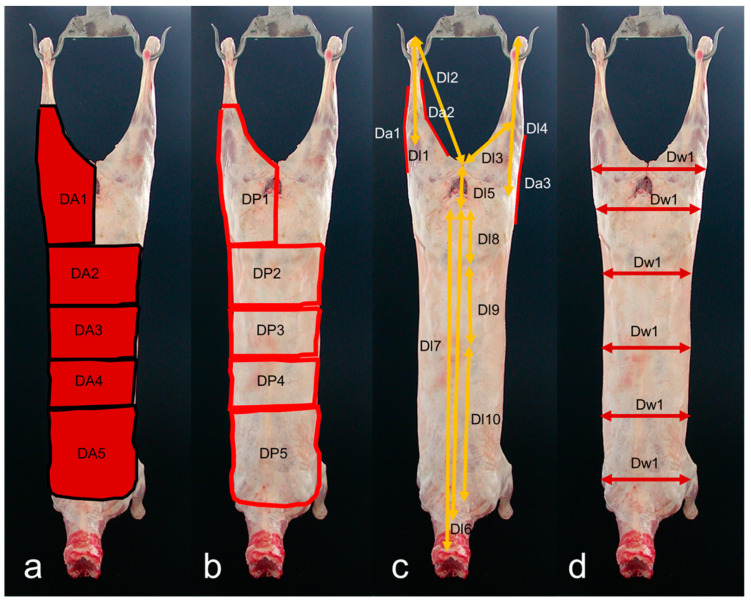
Dorsal side view of light lamb carcass showing areas (**a**), perimeters (**b**), lengths and angles (**c**), and widths (**d**) measurements. For a brief measurement description please see Table 1.

To ensure scale accuracy, two red dots were projected onto each carcass using parallel lasers (wavelength: 650 nm) mounted on a frame with predetermined spacing. These dots served as reference points for scale-bar purposes. The captured images were then transferred to a computer for analysis. Image analysis was performed using Fiji software (ImageJ 1.49u) developed by Rasband [16]. A total of 50 VIA measurements were recorded, comprising 21 from lateral view images (Figure 1a–d) and 29 from dorsal view images (Figure 2a–d). These measurements included areas, perimeters, lengths, angles, and widths taken from various regions of the carcass. In the present study, we build upon the documented measurements of length, area, width, and angle from prior research utilizing VIA systems. Specifically, insights from studies conducted by Batista et al. [17], Ngo et al. [18], Oliver et al. [19], and Rius-Vilarrasa et al. [20] have informed our approach and methodology.

### 2.3. Carcass Jointing and Dissection

Following the delineation by Santos et al. [21], the half-carcasses underwent division into six distinct cuts: neck, shoulder, breast, rib, loin, and leg. Subsequently, employing the methodology outlined by Panea et al. [22], all cuts were meticulously dissected into lean, fat (subcutaneous and intermuscular fat), and bone components within a controlled room environment ranging from 15 to 20 °C.

### 2.4. Models and Statistical Analysis

A comprehensive descriptive statistical analysis was conducted, encompassing the determination of mean, standard deviation, range, as well as the coefficient of variation for carcass characteristics such as CCW and weight, and percentage of muscle, subcutaneous fat, intermuscular fat, total fat, and bone, along with all VIA measurements.

For predictive modeling, K-fold stepwise regression analyses were employed to predict muscle, subcutaneous fat, intermuscular fat, total carcass fat, and bone weights and percentages. These models utilized either CCW plus VIA measurements or solely VIA measurements as independent variables. The accuracy of these predictions was assessed through the K-fold coefficient of determination (K-fold-R^2^), while the precision of the prediction models was evaluated using the residual mean square error (RMSE). The value of 0.05 was used as *p*-value threshold. Furthermore, the overall predictive ability of the k-fold cross-validation models was gauged through the ratio of percent deviation (RPD), which is the ratio of the standard deviation of the reference values to the RMSE of the validation [23]. All statistical procedures were executed using JMP software version 17 (SAS Institute, Cary, NC, USA).

## 3. Results

Table 1 summarizes the descriptive statistics (mean, standard deviation, range, and coefficient of variation) for cold carcass weight, weight, and percentage of tissues and VIA measurements obtained with lateral and dorsal views of the light lamb carcasses.

The average cold carcass weight recorded was 4.52 kg. When considering the amount of carcass tissues, greater variation was evident, with coefficients of variation ranging from 22.9% for bone, 29.2% for muscle, 38.9% for intermuscular fat, to 58.6% for subcutaneous fat. In terms of VIA measurements, area measurements exhibited the most significant variation, with relatively close coefficients of variation (CV) observed for both lateral and dorsal views (20.0% < CV < 25.4% and 18.0% < CV < 25.0%, respectively). Conversely, angle measurements displayed the least variation for both lateral and dorsal views (3.5% < CV < 6.1% and 3.1% < CV < 3.4%, respectively).

### 3.1. Prediction of Carcass Tissues Weight in the Carcass

The predictors, coefficients of determination (K-fold-R^2^), root mean square error (RMSE), and the ratio of prediction to deviation (RPD) for the best models to estimate the weight of carcass tissues are presented in Table 2.

The stepwise regression analysis using CCW and VIA measurements showed that the best prediction equations explained most of the variation observed in the weight of all carcass tissues (0.83 < K-fold-R^2^ < 0.98). The highest accuracy was observed for muscle, using model 1 (based on dorsal + lateral view data), and the lowest was for subcutaneous fat, also using model 1. Regardless of using model 1, model 2 (based on dorsal view data), or model 3 (based on lateral view data), CCW was the first independent variable in the best models for all tissues, which always included at least one VIA measurement. The number of VIA measurements included in the best prediction equation for each tissue was quite similar for the three models, except in the case of muscle, with the best prediction equation (model 1) including eight VIA measurements as independent variables, while models 2 and 3 only included one VIA measurement. For each tissue, the accuracy of the estimates obtained with the three models was quite similar, although the precision of the estimates was consistently higher for model 1—the largest difference was observed for bone (K-fold-R^2^ = 0.95 and RMSE = 21.76 for model 1; K-fold-R^2^ = 0.91 and RMSE = 28.20 for model 2, Table 2). For each tissue, concerning VIA measurements, there was little correspondence between the independent variables included in model 1 and the independent variables included in models 2 and 3. In the case of muscle, there were several independent variables from the dorsal and lateral view, but none of the ones included in models 2 and 3 (DP3 and LA1, respectively). For subcutaneous fat and bone, the first independent variables in model 1 corresponded to the first independent variables of models 2 and 3, but for muscle and intermuscular fat that did not happen. In the case of muscle, the first independent variable included in model 3 (Lw6; Table 3) was not even included in model 1. Besides CCW, model 1 included only lateral view data for intermuscular fat, while bone included more dorsal view data than lateral view data, and muscle included a similar amount of dorsal and lateral view data. When CCW was not included in the stepwise regression analysis the best prediction equations still explained a very large amount of the variation observed in the weight of all carcass tissues (0.74 < K-fold-R^2^ < 0.98; Table 3), regardless of the model used. For each tissue, model 1 included one to three more independent variables than the next best model, which was always model 2. Model 1 showed consistent but just slightly higher accuracy (0.89 < K-fold-R^2^ < 0.98, against 0.86 < K-fold-R^2^ < 0.94, for model 2) and higher precision, the largest difference being observed for muscle (K-fold-R^2^ = 0.98 and RMSE = 56.64, for model 1; K-fold-R^2^ = 0.94 and RMSE = 84.22, for model 2).

As for the analysis including CCW, the muscle was the tissue most accurately estimated (0.89 < K-fold-R^2^ < 0.98). However, excluding CCW from the analysis, intermuscular fat estimates became the ones showing the lowest accuracy (0.82 < K-fold-R^2^ < 0.89) except in the case of model 3, with the subcutaneous fat estimates remaining the ones showing the lowest accuracy (K-fold-R^2^ = 0.74). For subcutaneous fat and bone, the first independent variables in model 1 corresponded to the first independent variables of models 2 and 3, but for muscle and intermuscular fat that did not happen. In the case of muscle, the first independent variable included in model 3 (Lw6; Table 3) was not even included in model 1. Across the different tissues, model 1 included more dorsal view data than lateral view data and more independent variables than models 2 and 3.

### 3.2. Prediction of Carcass Tissues Percentage in the Carcass

The predictors, coefficients of determination (K-fold-R^2^), root mean square error (RMSE), and the ratio of prediction to deviation (RPD) for the best models to estimate the percentage of carcass tissues are presented in Table 4.

The models for the prediction of carcass tissue percentages explained a much smaller amount of the variation observed than the equivalent models for the prediction of carcass tissue weights, except in the case of model 1 for subcutaneous fat, which showed K-fold-R^2^ = 0.83 and 0.78, respectively, for subcutaneous fat weight and subcutaneous fat percentage (Table 2 and Table 4). Still, with the exception of model 2 for muscle and intermuscular fat, the best prediction equations showed moderate to high accuracy (0.41 < K-fold-R^2^ < 0.78; Table 4) in predicting the percentage of the different carcass tissues. The highest accuracy was observed for subcutaneous fat, using model 1 (based on dorsal + lateral view data), and the lowest was for muscle, using model 2 (K-fold-R^2^ = 0.16; Table 4). Although CCW was the first independent variable in some of the best prediction equations (model 3 for all tissues except muscle and model 1 for bone), it was not included in most of the best prediction equations (Table 4). The number of VIA measurements included in the best prediction equation for each tissue was particularly larger in the case of model 1 for subcutaneous fat, total fat, and bone, in the latter case including CCW as well, particularly in the case of muscle (K-fold-R^2^ = 0.45; RMSE = 1.77), subcutaneous fat (K-fold-R^2^ = 0.78; RMSE = 0.84), and bone (K-fold-R^2^ = 0.72; RMSE = 1.00). Concerning VIA measurements, the first independent variables of models 2 and 3 were included in model 1, except in the case of the equations for muscle and intermuscular fat, which did not include the first independent variables of model 2 and included mainly lateral view data as independent variables, unlike model 1 for each of the other tissues, which included mainly dorsal view data. (Table 4). For those tissues with the best prediction equations including CCW (intermuscular fat, subcutaneous fat), when CCW was removed from the stepwise analysis, the best prediction equations showed poor to moderate accuracy (0.37 < K-fold-R^2^ < 0.46).

## 4. Discussion

Concerning the prediction of carcass tissue weight or carcass tissue percentage, regardless of the methodology/technology applied, the literature consistently shows body weight or carcass weight as the strongest single predictor, the question being if prediction accuracy is significantly increased using such different methodologies/technologies. In line with this, the present study showed CCW as the first independent variable included in the best models for the prediction of carcass tissue weights. However, for carcass tissue percentages, with the exception of bone, when the stepwise analysis used CCW data, CCW was included only in the best models without dorsal VIA data. Such a finding agrees with Araújo et al. [24]. In fact, working with hair sheep lambs, castrated males, from commercial herds, finished in confinement and slaughter in a range of weight from 21 to 49 kg, Araújo et al. [24] showed better estimates of CCW using dorsal view data than lateral view data and related this to the fact that in the dorsal region it is possible to use measurements that represent a large part of the carcass, with large deposition of musculature and adipose tissue. Horgan et al. [25] had already studied the contribution of dorsal and lateral view data for the best prediction equations using castrated male lambs reared at grass, weaned at an average age of 12 weeks, and slaughtered when they reached a target live weight of 42 kg. They showed that in collecting dorsal and lateral view data, most of the data included in the best prediction equations were supplied by the dorsal plan and, using just dorsal or lateral view data in the multiple regression analysis, most carcass traits were better predicted in the former case. The present study showed the same trend for carcass tissue weights when CCW was not included in the analysis, with model 1 including more dorsal view data than lateral view data as independent variables and model 2 showing higher accuracy than model 3, for all carcass tissues. Not so much when CCW was included in the analysis, nor when carcass tissues were predicted as a percentage of CCW, suggesting that, when the effect of CCW is accounted for, either including CCW as an independent variable or expressing carcass tissues of a percentage of CCW, lateral view data provide more complementary information than dorsal view data. The little correspondence observed, in most cases, between the independent variables included in model 1 and the independent variables included in models 2 and 3, for each carcass tissue, strengthens the idea that the complementarity of the information given by each independent variable for the best prediction equation is more valuable than the individual information of each independent variable. It is not possible to make a direct comparison between the present study with previous studies using VIA data since different studies used different linear measurements, area measurements, or even color, and some refer to saleable meat, which included a variable amount of fat and, sometimes, bone [4,12]. Also, factors such as breed, weight at slaughter, and feeding background have a strong effect on carcass composition. Previous studies on assessment of lamb carcasses by video image analysis have been conducted with quite different experimental populations, such as Scottish Mule Χ Suffolk male castrated lambs reared at grass, weaned at an average age of 12 weeks and slaughtered when they reached a target live weight of 42 kg [25], rams, ewes, and wethers of either wool-type (Rambouillet, Targhee) or large-frame meat-type (Suffolk, Hampshire), with a carcass weight of 24.3–27.2 kg [26], lamb carcasses reflecting the extreme range of variation in carcass traits experienced in commercial facility with hot carcass weight (HCW) ≤ 29.48 kg (light-muscled carcasses), HCW = 29.94 kg to 34.02 (medium-muscled carcasses) and HCW ≥ 34.47 kg (heavy-muscled carcasses) [27] and CCW = 21.22 kg to 54.34 kg [28], mixed sex and breed types selected to cover as far as possible the full spectrum of lambs slaughtered in Australia, with HCW = 13.6 to 34.0 kg [29] and purebred ram and ewe lambs of the Icelandic breed, with HCW = 7.2 to 26.8 kg [30]. Still, the present results compare well with results from such previous studies that showed R^2^ = 0.95 for saleable meat weight [25], R^2^ = 0.99 for lean meat weight [5], R^2^ = 0.58 for fat weight [25], 0.16 < R^2^ < 0.71 for saleable meat yield ([25,26,27,31], 0.48 < R^2^ < 0.74 for fat yield ([25,27,28], R^2^ = 0.60 for boneless saleable meat yield [29] and 0.52 < R^2^ < 0.58 for lean meat wield [29,30], particularly given the fact that the carcass weight the animals now used (mean CCW = 4.5 kg) was much smaller than the carcass weight of the animals used in such studies. The largest number of VIA measurements used as independent variables in the best prediction equations now obtained (nine VIA measurements with model 1, for subcutaneous fat percentage, excluding CCW from the analysis) is well in the range of the number of VIA measurements in the prediction equations developed in the same studies, (six to eight VIA measurements in most cases). Although there was a clear trend for model 1 to show higher accuracy and precision across all carcass tissues, considering the possibility of implementation of a VIA system under commercial abattoir conditions, this has to be balanced with the fact that model 1 also includes more VIA measurements as independent variables and implies the acquisition of images from two view plans instead of just one as in the case of models 2 and 3. If a larger number of VIA measurements to be collected may not be a problem with an automated system and the software resources currently available, creating the conditions to collect dorsal and lateral view data without interfering with the high speed of a slaughter line may not be practical. According to Viscarra Rossel et al. [32] most of the best prediction equations obtained in the present study for carcass tissue weights can be qualified as having excellent predictive value, presenting RPD > 2.5. The exceptions were the equations obtained with model 3 for fat weights when CCW was not included in the stepwise analysis. Even so, the equations for intermuscular fat and total fat showed very good predictive value (2.0 < RPD < 2.5) and the equation for subcutaneous fat showed good predictive value (1.8 < RPD < 2.0). Although the best equations for the prediction of the percentage of carcass tissues explained a considerably lower amount of the variation observed than the best equations for the prediction of carcass tissue weights, only the ones for the prediction of muscle percentage and model 2 for prediction of intermuscular fat percentage showed poor predictive value (1.0 < RPD < 1.4). The best equation for the prediction of subcutaneous fat percentage based on model 1 even showed excellent predictive value (RPD = 2.5).

## 5. Conclusions

This study not only confirms results from previous studies showing that information extracted from images of lamb carcasses can be used to explain most of the variation observed in carcass composition but also suggests that the same applies to light lamb carcasses, providing an objective means in conjunction of CCW, to have a value-based payment system that leads to a fair payment to the producer, according to the preference of the consumers. VIA data obtained from dorsal and lateral views provided higher accuracy and precision estimates than dorsal or lateral view data across all carcass tissues. However, considering the possibility of implementing a VIA system under commercial abattoir conditions, the need to reduce to a minimum the interference with the production chain may justify the option for using dorsal or lateral view data only. This needs further analysis, as well as additional studies, to strengthen the present results on light lamb carcasses.

## Figures and Tables

**Table 1:** Mean (standard deviation—sd), range, and coefficient of variation (CV) for cold carcass weight (CCW), weight and percentage of carcass tissues, and VIA measurements obtained in lateral and dorsal views (n = 55).

Traits	Abbreviation	Description	Mean (sd)	Range	CV (%)
CCW (g)			4522 (1336)	2162–7622	29.6
Carcass composition					
Carcass tissues (g)	M	Muscle	1169.5 (341.4)	546.9–1945.6	29.2
	SF	Subcutaneous fat	131.0 (76.8)	11.8–359.4	58.6
	IF	Intermuscular fat	236.4 (91.9)	96.0–475.7	38.9
	TF	Total fat	367.4 (166.0)	107.9–834.9	45.2
	B	Bone	447.8 (102.5)	259.6–683.3	22.9
Carcass tissues (%)	pM	Percentage muscle	61.1 (2.4)	56.4–69.7	4.0
	pSF	Percentage subcutaneous fat	6.1 (2.1)	1.1–10.7	34.2
	pIF	Percentage intermuscular fat	10.9 (1.7)	5.9–13.9	15.2
	pTF	Percentage total fat	17.0 (3.5)	7.0–24.7	20.4
	pB	Percentage bone	21.9 (2.1)	17.4–26.0	9.7
Lateral view measurements					
Length (cm)	Ll1	Leg length	30.8 (3.4)	23.0–38.0	11.0
	Ll2	Lateral thoracolumbar length	39.2 (4.3)	29.7–47.0	10.9
	Ll3	Length between the calcaneus and the greater tubercle of humerus	72.7 (6.9)	55.3–86.4	9.5
	Ll4	Forearm length	26.9 (3.2)	18.9–32.4	12.1
Width (cm)	Lw1	Thinnest leg width	9.7 (1.1)	7.2–12.0	11.6
	Lw2	Largest leg width	10.3 (1.3)	7.7–13.0	12.7
	Lw3	Minimum waist width	9.4 (1.2)	7.1–12.7	12.6
	Lw4	Maximum waist width	13.2 (1.7)	9.2–16.6	13.0
	Lw5	Maximum thoracic width	17.1 (2.1)	13.1–20.9	12.1
	Lw6	Widest part of the chest	17.4 (2.0)	13.1–22.7	11.5
Angle (θ)	La1	Lateral leg angle 1	142.6 (5.7)	129.8–153.9	4.0
	La2	Lateral leg angle 2	160.5 (5.6)	149.2–172.8	3.5
	La3	Lateral leg angle 3	154.6 (9.5)	136.0–178.9	6.1
Area (cm^2^)	LA1	Lateral leg area	185.0 (42.8)	107.7–271.6	23.1
	LA2	Loin area	163.1 (39.8)	97.8–270.6	24.4
	LA3	Forequarter area	337.7 (67.5)	204.2–471.3	20.0
	LA4	Lateral shoulder area	140.3 (35.6)	83.5–260.7	25.4
Perimeter (cm)	LP1	Lateral leg perimeter	58.6 (7.1)	44.0–70.7	12.2
	LP2	Loin perimeter	50.8 (7.9)	14.0–65.7	15.5
	LP3	Forequarter perimeter	69.8 (10.4)	15.3–83.2	14.9
	LP4	Lateral shoulder perimeter	48.7 (8.2)	11.4–67.5	16.8
Dorsal view measurements				-	
Length (cm)	Dl1	Minimum leg length	16.3 (3.8)	8.6–27.0	23.3
	Dl2	Length between the perineum and the tarsometatarsal joint	19.3 (2.6)	11.4–25.0	13.4
	Dl3	Length between the perineum and the femorotibial joint	6.4 (0.8)	4.9–8.8	12.5
	Dl4	Maximum leg length	24.5 (3.8)	6.1–33.8	15.5
	Dl5	Length between the basis of tail and the perineum	9.1 (1.8)	6.0–14.3	20.0
	Dl6	Length between the basis of the neck and the basis of tail	49.7 (4.8)	41.2–61.4	9.6
	Dl7	Length between the basis of tail and the atlas	62.0 (5.7)	48.9–77.1	9.2
	Dl8	Sacrum length	8.6 (1.3)	5.9–11.9	14.7
	Dl9	Lumbar spine length	18.9 (3.0)	10.2–26.3	15.9
	Dl10	Thoracic spine length	29.5 (4.2)	19.9–38.9	14.1
Width (cm)	Dw1	Minimum leg width	17.5 (1.5)	14.2–21.8	8.4
	Dw2	Maximum leg width	13.0 (1.4)	10.4–17.4	10.9
	Dw3	Minimum saddle width	9.7 (1.2)	7.9–13.0	12.7
	Dw4	Maximum chest width	10.6 (1.4)	7.6–14.3	13.3
	Dw5	Minimum chest width	9.8 (1.1)	7.0–12.0	11.2
	Dw6	Maximum shoulder width	10.9 (1.3)	7.6–13.3	11.9
Angle (θ)	Da1	Dorsal leg angle 1	164.1 (5.6)	150.2–174.0	3.4
	Da2	Dorsal leg angle 2	122.3 (4.0)	113.7–130.7	3.3
	Da3	Dorsal leg angle 3	167.7 (5.2)	157.3–179.7	3.1
Area (cm^2^)	DA1	Dorsal leg area	131.0 (23.5)	89.8–203.0	18.0
	DA2	Lumbar area	104.4 (26.8)	55.7–199.8	25.7
	DA3	Thoracolumbar area	105.1 (26.3)	58.4–171.9	25.0
	DA4	Thoracic area	101.9 (23.6)	57.4–189.8	23.2
	DA5	Dorsal shoulder area	136.8 (33.7)	77.2–242.1	24.6
Perimeter (cm)	DP1	Dorsal leg perimeter	55.9 (4.9)	45.3–71.8	8.8
	DP2	Lumbar perimeter	42.3 (5.2)	31.9–60.2	12.3
	DP3	Thoracolumbar perimeter	40.3 (4.9)	29.8–53.1	12.2
	DP4	Thoracic perimeter	40.1 (4.7)	30.4–54.6	11.6
	DP5	Dorsal shoulder perimeter	46.2 (5.7)	35.6–62.1	12.3

**Table 2:** Equations and corresponding coefficient of determination (K-fold-R^2^), root mean square error (RMSE), and ratio of prediction to deviation (RPD) for prediction of the weight of carcass tissues in lamb carcasses for stepwise analysis with CCW (n = 55).

Tissue		Dorsal + Lateral	(Model 1)	Dorsal	(Model 2)	Lateral	(Model 3)
Muscle (g)	Intercept	−1570.57		−152.25		−73.05	
	Independent variables	0.128	CCW	0.23	CCW	0.197	CCW
		−7.293	Ll2	7.004	DP3	1.891	LA1
		10.052	Ll3				
		47.455	Lw3				
		3.72	La1				
		−6.278	Dl2				
		4.775	Dl11				
		46.776	Dw3				
		4.715	DP2				
	k-fold-R^2^	0.98		0.95		0.97	
	RMSE	42.05		63.46		54.19	
	RPD	8.12		5.38		6.3	
IF (g)	Intercept	406.761		−205.125		347.615	
	Independent variables	0.058	CCW	0.033	CCW	0.052	CCW
		−4.012	Ll1	15.509	Dl3	−3.121	La1
		−2.93	La1	1.47	DA1	0.619	LA2
		0.679	LA2				
	k-fold-R^2^	0.88		0.85		0.88	
	RMSE	28.82		34.28		29.98	
	RPD	3.19		2.68		3.07	
SF (g)	Intercept	45.155		−239.455		8.721	
	Independent variables	0.044	CCW	0.016	CCW	0.063	CCW
		−4.843	Ll1	−2.537	Dl4	−6.061	Ll4
		−2.392	Ll4	14.036	Dw3		
		−3.775	Dl7	1.722	DA1		
		11.523	Dw3				
		1.699	DA1				
	k-fold-R^2^	0.83		0.85		0.84	
	RMSE	24.07		27.61		29.5	
	RPD	3.19		2.78		2.6	
TF (g)	Intercept	388.234		−46.719		614.243	
	Independent variables	0.073	CCW	0.034	CCW	0.117	CCW
		−12.861	Ll1	24.227	Dl3	−9.676	Ll1
		−3.456	La1	−5	Dl4	−4.361	La1
		0.799	LA2	4.786	Dl10	0.885	LA2
		0.363	LA3	27.359	Dw3		
		2.171	DA1	−3.693	Da1		
				3.275	DA1		
	k-fold-R^2^	0.91		0.89		0.88	
	RMSE	45.81		49.72		50.42	
	RPD	3.62		3.34		3.29	
Bone (g)	Intercept	−244.683		−271.71		−58.374	
	Independent variables	0.04	CCW	0.039	CCW	0.055	CCW
		22.367	Lw1	5.022	Dl7	26.264	Lw1
		−16.285	Dl3	−20.496	Dw4		
		3.612	Dl6	24.96	Dw5		
		−17.319	Dw4	5.14	DP3		
		19.553	Dw5				
		5.189	DP3				
	k-fold-R^2^	0.95		0.91		0.92	
	RMSE	21.76		28.2		27.63	
	RPD	4.71		3.63		3.71	

CCW = Cold carcass weight; IF = intermuscular fat; SF = subcutaneous fat; TF = total fat; Ll2 = thoracolumbar length; Ll3 = length between the calcaneus and the greater tubercle of humerus; Lw3 = minimum waist width; La1 = lateral leg angle; Dl2 = length between the perineum and the tarsometatarsal joint; Dl11 = thoracic spine length; Dw3 = minimum saddle width; DP2 = lumbar perimeter; DP3 = thoracolumbar perimeter; LA1 = lateral leg area; Ll1 leg length: LA2 = loin area; Ll4 = forearm length; Dl7 = cervical spine length; DA1 = dorsal leg area; Dl3 = length between the perineum and the femorotibial joint; Dl4 = maximum leg length; LA3 = forequarter area; Dl10 = thoracic spine length; Da1 = dorsal leg angle 1; Lw1 = thinnest leg width; Dl6 = length between the basis of the neck and the basis of tail; Dw4 = maximum chest width; Dw5 = minimum chest width.

**Table 3:** Equations and corresponding coefficient of determination (K-fold-R^2^), root mean square error (RMSE), and ratio of prediction to deviation (RPD) for prediction of the weight of carcass tissues in lamb carcasses for stepwise analysis without CCW (n = 55).

Tissue		Dorsal + Lateral (Model 1)		Dorsal (Model 2)		Lateral (Model 3)	
Muscle	Intercept	−1259.646		−1281.543		−828.65	
	Independent variables	10.524	DA2	18.335	Dl7	66.034	Lw6
		7.022	Dw3	70.866	Dw3	4.598	LA1
		6.932	LA1	5.99	DA2		
		2.049	Ll4				
		1.417	Dl2				
		1.083	Dl11				
	K-fold-R^2^	0.98		0.94		0.88	
	RMSE	56.642		84.222		122.901	
	RPD	6.03		4.05		2.78	
IF	Intercept	−164.563		−455.011		97.734	
	Independent variables	−2.22	La1	16.103	Dl3	5.573	Ll2
		0.542	LA2	4.975	Dl7	16.602	Lw6
		4.236	Dl7	2.139	DA1	−3.683	La1
		9.573	Dw1			0.964	LA2
		1.522	DA1				
	K-fold-R^2^	0.89		0.86		0.82	
	RMSE	31.416		35.577		40.7	
	RPD	2.93		2.58		2.26	
SF	Intercept	−32.639		55.649		113.112	
	Independent variables	10.248	DA1	11.553	DA1	−6.682	Ll1
		5.954	Dw3	5.898	Dw3	1.531	LA1
		5.123	Ll1	3.438	Da1	0.578	LA2
		4.052	Da1	2.451	Dl4	0.716	LA3
		2.455	Lw3	1.935	Dl9	−6.75	LP1
		2.338	Dl7	1.487	Da3		
		1.843	DP5				
		1.501	LP3				
		1.479	Dl9				
	K-fold-R^2^	0.94		0.91		0.74	
	RMSE	20.598		23.978		41.358	
	RPD	3.73		3.2		1.86	
TF	Intercept	−134.537		−839.286		41.391	
	Independent variables	−10.169	Ll1	12.691	DA1	−12.034	Ll1
		1.926	LP3	3.805	Dw3	11.758	Ll2
		−4.516	Dl4	2.336	Da1	44.735	Lw6
		24.446	Dw1	1.81	Dl4	−5.603	La1
		30.785	Dw5	1.674	Dl9	1.582	LA2
		−3.988	Da1				
		5.482	DA1				
	K-fold-R^2^	0.92		0.91		0.80	
	RMSE	49.017		52.401		77.904	
	RPD	3.39		3.17		2.13	
Bone	Intercept	−24.68		−351.21		−250.958	
	Independent variables	5.905	Dl7	5.244	Dl7	6.53	Ll2
		4.241	Ll2	2.668	DP3	20.198	Lw1
		2.272	DP3	2.213	Dw5	0.906	LA1
		2.159	Da2	2.1	Dl12	0.486	LA2
		1.804	LA1	1.523	Dw4		
		1.634	Dl2				
	K-fold-R^2^	0.94		0.92		0.89	
	RMSE	25.864		30.175		34.997	
	RPD	3.96		3.40		2.93	

CCW = cold carcass weight; IF = intermuscular fat; SF = subcutaneous fat; TF = total fat; DA2 = lumbar area; Dw3 = minimum saddle width; LA1 = lateral leg area; Ll4 = forearm length; Dl2 = length between the perineum and the tarsometatarsal joint; Dl11 = thoracic spine length; Dl7 = cervical spine length; Lw6 = widest part of the chest; La1 = lateral leg angle 1; LA2 = loin area; Dw1 = minimum leg width; DA1 = dorsal leg area; Ll1 leg length: Dl3 = length between the perineum and the femorotibial joint; Ll2 = thoracolumbar length; Da1 = dorsal leg angle 1; Dl4 = maximum leg length; LA3 = forequarter area; Lw3 = minimum waist width; Dl9 = lumbar spine length; LP1 = lateral leg perimeter; Da3 = dorsal leg angle 3; DP5 = dorsal shoulder perimeter; LP3 = forequarter perimeter; Dw5 = minimum chest width; DP3 = thoracolumbar perimeter; Da2 = dorsal leg angle 2; Lw1 = thinnest leg width; Dl12 = cervical spine length; Dw4 = maximum chest width.

**Table 4:** Equations and corresponding coefficient of determination (K-fold-R^2^), root mean square error (RMSE), and ratio of prediction to deviation (RPD) for prediction of the percentage of carcass tissues in lamb carcasses for stepwise analysis with CCW (n = 55).

Tissue		Dorsal + Lateral (Model 1)		Dorsal (Model 2)		Lateral (Model 3)	
Muscle (%)	Intercept	38.948		59.928		37.757	
	Independent variables	0.594	Ll1	0.214	Dl4	0.594	Ll1
		−0.385	Ll2	−0.179	Dl10	−0.385	Ll2
		0.171	La1	0.733	Dw6	0.171	La1
		−0.026	LA2	−0.051	DA1	−0.026	LA2
	k-fold-R^2^	0.46		0.16		0.41	
	RMSE	1.77		2.177		1.776	
	RPD	1.36		1.1		1.35	
IF (%)	Intercept	11.671		3.621		23.099	
	Independent variables	−0.222	Ll1	0.896	Dl3	0.001	CCW
		0.216	Ll2	−0.129	Dl4	−0.231	Ll1
		−0.09	La1	0.161	Dl10	0.165	Ll2
		−0.033	LA1			−0.097	La1
		0.024	LA2			−0.032	LA1
		0.203	Dl7			0.022	LA2
	k-fold-R^2^	0.50		0.23		0.51	
	RMSE	1.089		1.385		1.07	
	RPD	1.56		1.23		1.50	
SF (%)	Intercept	10.3		−1.698		8.06	
	Independent variables	−0.328	Ll1	−0.178	Dl4	0.002	CCW
		−0.126	Dl4	0.524	DW3	−0.308	Ll1
		−0.239	Dl5	0.053	DA1		
		0.737	DW3				
		−0.11	Da1				
		0.078	DA1				
		−0.084	DA4				
		0.403	DP4				
		0.092	DP5				
	k-fold-R^2^	0.78		0.59		0.56	
	RMSE	0.841		1.271		1.329	
	RPD	2.5		1.65		1.64	
TF (%)	Intercept	51.799		−18.547		34.396	
	Independent variables	−0.496	Ll1	−0.329	Dl4	0.003	CCW
		−0.104	La1	0.242	Dl10	−0.552	Ll1
		−0.26	Dl4	0.186	Da2	0.925	Lw6
		−0.317	Dl5	0.105	DA1	−0.144	La1
		0.218	Dl9			−0.051	LA1
		1.188	Dw3				
		−0.168	Da1				
		0.126	DA1				
	k-fold-R^2^	0.63		0.49		0.58	
	RMSE	1.827		2.264		2.073	
	RPD	1.92		1.55		1.61	
Bone (%)	Intercept	44.53		32.965		23.029	
	Independent variables	0.001	CCW	0.119	Dl4	−0.002	CCW
		0.271	Ll2	−0.244	DW3	0.161	Ll2
		−0.011	LA3	−0.454	Dw6		
		0.127	Dl4	−0.051	DA1		
		−0.358	Dw1				
		−0.612	Dw6				
		−0.107	Da2				
		−0.043	DA1				
	k-fold-R^2^	0.72		0.64		0.62	
	RMSE	0.996		1.210		1.288	
	RPD	2.11		1.74		1.71	

CCW = cold carcass weight; IF = intermuscular fat; SF = subcutaneous fat; TF = total fat; Ll1 = leg length: Ll2 = thoracolumbar length; Dl4 = maximum leg length; La1 = lateral leg angle 1; Dw6 = maximum shoulder width; Dl10 = Y; DA1 = leg area; Dl9 = lumbar spine length; LA2 = loin area; LA1 = lateral leg area; Dw3 = minimum saddle width; Dl5 = length between the basis of tail and the perineum; LA3 = forequarter area; Da1 = dorsal leg angle 1; Dl3 = length between the perineum and the femorotibial joint; DA4 = thoracic area; DP4 = thoracic perimeter; DP5 = dorsal shoulder perimeter; Da2 = dorsal leg angle 2; Lw6 = widest part of the chest; Dw1 = minimum leg width.

## Data Availability

Data are available upon request to the corresponding author and with permission from study participants.
